# Optimized CNNs to Indoor Localization through BLE Sensors Using Improved PSO

**DOI:** 10.3390/s21061995

**Published:** 2021-03-12

**Authors:** Danshi Sun, Erhu Wei, Zhuoxi Ma, Chenxi Wu, Shiyi Xu

**Affiliations:** 1 School of Geodesy and Geomatics, Wuhan University, Wuhan 430079, China; 2017102140028@whu.edu.cn; 2 Xi’an Division of Surveying and Mapping, Xi’an 710054, China; mzhx_1991@163.com; 3 BGI Engineering Consultants Ltd., Beijing 100038, China; tracywu0302@163.com; 4 Beijing Satellite Navigation Center, Beijing 100094, China; sarah_741@163.com

**Keywords:** BLE sensors, signal strength indicator, indoor positioning, bat algorithm, convolutional neural network

## Abstract

Indoor navigation has attracted commercial developers and researchers in the last few decades. The development of localization tools, methods and frameworks enables current communication services and applications to be optimized by incorporating location data. For clinical applications such as workflow analysis, Bluetooth Low Energy (BLE) beacons have been employed to map the positions of individuals in indoor environments. To map locations, certain existing methods use the received signal strength indicator (RSSI). Devices need to be configured to allow for dynamic interference patterns when using the RSSI sensors to monitor indoor positions. In this paper, our objective is to explore an alternative method for monitoring a moving user’s indoor position using BLE sensors in complex indoor building environments. We developed a Convolutional Neural Network (CNN) based positioning model based on the 2D image composed of the received number of signals indicator from both x and y-axes. In this way, like a pixel, we interact with each 10 × 10 matrix holding the spatial information of coordinates and suggest the possible shift of a sensor, adding a sensor and removing a sensor. To develop CNN we adopted a neuro-evolution approach to optimize and create several layers in the network dynamically, through enhanced Particle Swarm Optimization (PSO). For the optimization of CNN, the global best solution obtained by PSO is directly given to the weights of each layer of CNN. In addition, we employed dynamic inertia weights in the PSO, instead of a constant inertia weight, to maintain the CNN layers’ length corresponding to the RSSI signals from BLE sensors. Experiments were conducted in a building environment where thirteen beacon devices had been installed in different locations to record coordinates. For evaluation comparison, we further adopted machine learning and deep learning algorithms for predicting a user’s location in an indoor environment. The experimental results indicate that the proposed optimized CNN-based method shows high accuracy (97.92% with 2.8% error) for tracking a moving user’s locations in a complex building without complex calibration as compared to other recent methods.

## 1. Introduction

The academic industry has shown immense interest in the field of the Internet of Things (IoT) because it can propose smart and innovative solutions. Extraordinary utilization of IoT in our routine lives and its regular use in several intelligent environments (e.g., smart industry, smart universities, smart homes, and smart cities) requires an extension of solutions for efficient and effective communication of IoT devices [1]. Keeping balanced requirements like range, connection density, latency, bandwidth, quality of service, and, endpoint cost is part of IoT networking [2,3]. Recent enhancements of IoT electronics, computing paradigms and protocols result in the evolution of wireless sensor networks (WSNs) having strong abilities for several applications in industrial, biomedical, civil, military, environmental, etc. WSN is made up of plenty of sensors with abilities to communicate, process data and sense [4,5].

The observation of paths and locations of individuals through indoor traversal is becoming a vital element of applications involving contextual information like the analysis of the workflow of a clinical environment [6,7] and representation of viral diseases [8,9]. Conventional methods like surveys are slow, incompetent and expensive, particularly when the observation is required all the time and numerous people are required to be traced at a time [10]. Although numerous technologies exist for localization [11,12], these are still far behind the performance of global positioning systems (GPS) in an outdoor environment [13]. Technologies based on radio frequency (RF) are usually used for indoor tracking, but these are affected by nearby sensors and transmitters, making them unreliable. Prior solutions were only examined in closed and controlled environments with a high cost of deployment [14]. Learning the properties of RF signals can aid in designing a better solution for indoor localization.

Existing RF-based techniques are not feasible in every environment. Also, the claim that Wi-Fi-based techniques are inappropriate for environments, such as hospitals, due to the unavailability of signals in certain areas is questionable because Wi-Fi has a longer range than Bluetooth low energy (BLE). Additional RF techniques based on indoor localization rely on Bluetooth low energy (BLE) and RFID [14,15]. BLE is a better option than RFID in multi-locations and multi-user applications due to the low consumption of power and low cost. Information of signal like travel time, direction, the relative strength of signal [16] is used in most of the RF-based techniques. RSSI techniques have a low implementation cost and are easy to implement. Earlier methods used slow algorithms requiring expensive calibration. For instance, methods relying on fingerprints will not operate properly if currently sensed data vary significantly from patterns used during training.

It is assumed that more powerful signals will be detected by the receiver of the nearest BLE beacons. Extra calibrations are required to remove signals from inaccurate locations in settings where signal interference may cause difficulties. A threshold rate will be used to manage outlier signals, signals having a value lower than the threshold rate will be removed by using a novel algorithm. Threshold value may vary from location to location.

Machine learning (ML) algorithms were proposed last year in various applications like energy [17,18,19], health [20,21], communication [17,22], agriculture [23,24] and so forth. It was achieved due to the following reasons: (1) growing computational abilities of microprocessors and circuits, and (2) access to big data offered by the internet. Unluckily, machine learning techniques demand transmission of all data gathered by sensors, which is a huge barrier in several cases, like in cases where data transmission demands data rates that cannot be neglected. In such cases, to ensure accurate communication of all wireless techniques with low power is not permissible.

Bio inspired methodologies make the data processing efficient in terms of power consumption [25,26] and computational power. It is expected that optimization algorithms will become closer to the sensor nodes for the local processing of data and transmission of less information to accuracy and precision of positioning and of execution and memory management.

Particle Swarm Optimization (PSO) is a commonly known socially inspired optimization algorithm. A community of particles, known as a swarm, fly and scan at a certain speed in a restricted range. PSO has been widely applied to solve real-time optimization problems. Since weight optimization during Convolutional Neural Network (CNN) is a major problem. Furthermore, the number of layers of CNN need to be decided at run time instead of predefined fix layers. For optimal parameter selection and to reduce the local minima problem, PSO is used widely in the similar applications

In this paper, we have proposed a novel CNN architecture optimized by improved PSO for the indoor positioning system. We adopt a neuro-evolution approach for developing CNN adaptively. For optimal parameter selection, PSO is used and further enhanced through dynamic inertia weight factor and social cognitive factors. For this, we convert BLE RSSI beacons data into 2-d images through x and y coordinates and evaluate proposed optimized CNN for indoor positioning through BLE RSSI signals. We compared proposed CNN with other ML and DL state of the art algorithms such as Decision Tree, K-Nearest Neighbor (KNN), Logistic Regression, Support Vector Machine (SVM), Artificial Neural Network (ANN), ANN with dropout, Deep Neural Network (DNN), DNN with dropout, and finally Multi Layer Perceptron (MLP).

The organization of the remainder of the paper is—related work along with systematic literature studies in the compact form are discussed in Section 2. Detail related to locating transmitters, system architecture and the proposed framework are given in Section 3 where data collection, experimental results, and analysis of simulations are enlisted in Section 4. We concluded the study in Section 5.

## 2. Related Work

A method based on the received number of signals indicator (RNSI) was proposed by the authors [27] to predict the location of a user. They experimented with their method in hospital and office settings. A comparison was made between RSSI and RNSI at several ranges from beacons of BLE. To experiment with the movement of a user, a user with a beacon was made to walk on a pre-defined path. Prediction made was ranked in terms of accuracy.

The authors of [28] carried out a study to propose a system for the accurate localization of users touring some cultural places or a museum. They expect that a BLE device is attached to the visitor that regularly transmits packets, those packets are captured by a receiver mounted inside the museum. The locator server is equipped with gathered packets that estimate a visitor’s location in the museum. The estimated position is achieved by using a feed-forward neural network, which is trained by using a non-linear least square algorithm. Their proposed method showed an accuracy of the estimated position under 1 m.

Another study based on iBeacon protocol has been carried out by authors [29]. They examined their method in three different environments in terms of frequency of barriers. The results gained showed that their algorithm beat the conventional algorithm particularly in settings with high or medium frequencies of barriers.

The authors propose an innovative data set [30]. They built their data set to facilitate a reliable and vast quantity of RSSI values gained from wearable and fixed BLE devices to experiment solutions working with distinct configurations comprising of social interactions and room possession along with tracking and localization.

Authors carried out a study [31] to suggest UILOC which is an unsupervised localization technique that utilizes Wi-Fi fingerprints, iBeacons and sensors of smartphone for precise and reliable indoor localization without any labor cost. At first, they compared their method with fingerprint-based techniques, their proposed method can generate a database of fingerprints automatically without site survey and then they implement the database in localization algorithm of fingerprints. After that, their proposed method will give the estimated location by using the pedestrian dead reckoning (PDR) technique.

Another work [32] has been proposed to analyze threats, opportunities, weaknesses and strengths to examine the current status of positioning methods of UWB. Their proposed study exhibits novel taxonomies, debates for more investigations about this problem from the research community, and analyze recent significant advancements. Similarly, the authors proposed online collaborative localization (OCLoc) scheme [33] for Online Collaborative Localization. In their work, they enhanced the RSSI strength through multiple requesting users.

A hierarchical indoor location (HiLoc) framework is proposed [34] to enhance radio surface location accuracy from crowdsourced samples. Tian et al. carried out a study [35] to deal with issues of AP optimization and placement of a beacon by suggesting a novel Cramer-Rao lower bound (CRLB) based heuristic differential evolution algorithm. Rather than utilizing the log distance path loss (LDPL) technique, they adopted the Motley-Keenan model to exhibit the impacts of comprehensive barriers in indoor settings.

A study [36] has been carried out to exhibit unstable characteristics of RSSI and a novel method was proposed comprising of three states, that is, invisible, retreating and approaching. They tested their method in the real world and illustrated that their method can predict Perceive Direction Information (PDI) by utilizing a Dense Neural Network while providing 95% accurate results even on untrained devices. After that, they introduced Monte Carlo Localization (MCL) algorithm that utilized PDI to gain highly accurate results.

A novel framework was proposed by authors [37] under conditions of non-line-of-sight (NLoS) by utilizing some knowledge of channel state information (CSI) gained from low power wide area (LPWA) radios. Their proposed method is an NLoS CSI classification-based framework utilizing deep learning (DL) and machine learning (ML) models. They illustrated their proposed framework which provided 98% accurate results by utilizing a multilayer neural network (MNN).

Pu et al. proposed a novel fingerprint-based location algorithm [38] to predict the location of the desired node by utilizing weighted and general algorithms of k-nearest neighbor. They trained grid points at 2 m intervals and the predicted error in position was about 1.8 m. Hence, their suggested model consumes low computations and provided satisfactory accuracy rates.

Authors proposed a [39] novel indoor localization framework offering accurate and fast positioning predictions. They utilized an Expectation-maximization (EM) algorithm for handling issues of varied mobile devices. Their proposed framework is best for exhibition environments as it enhanced the accuracy rate of localization in different mobile devices while implementing fingerprint strategy.

In [40] the authors analyzed BLE-based algorithms for indoor localization. They ensembled and implemented various localization algorithms like Transliteration Localization, Weighted Centroid Localization, Centroid Localization, and Proximity Localization. They also executed and suggested fuzzy logic based technique for choosing the fittest algorithm based on room size, signal strength and quantity of beacons.

Hou et al. brought out a study [41] for helping patients in finding their relevant clinics or departments which in terms required an indoor localization technique offering accuracy of room level. They studied the AOA-based approach to aid patients in finding their ways in hospitals wherever access points of Wi-Fi are mounted. Their proposed framework showed promising results.

The authors introduced a novel framework [42] for indoor localization by utilizing the smartphone of the user. Their proposed method of automatic and real-time method of data set construction offers low cost and efficient construction of data set. Their proposed model can also be applied to all sorts of signal processing of time series sensors. They examined their framework on an android app.

A novel framework [43] was proposed for modeling RSSI’s non-Gaussian properties to sustain the load of computations. The outcomes of simulations based upon gathered signals of RSSI guaranteed success of Wasserstein Distance (WD-based) Gaussian Sum Filter (GSF) framework ensembled standard equivalents. Their investigational results exhibit that their method gives more accurate results than conventional algorithms.

Girgensohn et al. carried out a study [44] to improve the ability to range technology to contribute beneficial predictions of location. Velocity was selected as a parameter of motion state. They exhibit useful enhancements accuracy of predicted indoor localization. Moreover, the real-world implementation provided valuable suggestions for upcoming IoT applications.

Another study has been carried out [45] to investigate BLE-based algorithms for indoor localization. They suggested RSSI of BLE beacons along with current beacon’s distance from point of the fingerprint to determine Euclidean distance for predicting location. Their gained outcomes fingerprint algorithm showed the best results by utilizing type 2 of fuzzy logic for indoor localization.

A study has been carried out [46] to propose a system for indoor localization based on BLE iBeacon by utilizing the fingerprint technique. They applied the k-nearest neighbors (KNN) algorithm to predict user location. To offer higher accuracy rates and a low computational cost they suggested utilizing lightweight vectors to train the algorithm of machine learning. Outcomes of their system provided useful predictions of the location of the user. Some studies suggested [47] GRU as deep learning model to test on such optimization problems.

An analysis was made by authors [48] to study the variations of BLE signals due to alteration of power levels in BLE transmission. To examine the consequences they organized a model for localization by utilizing the following methods, that is, Minimum Mean Square Error (MMSE) and Centroid Approximation (CA).

Turgut et al. proposed a system [49] for indoor localization to offer a minimum cost of infrastructure and utilizing technologies of the present building. They generated the signal map by utilizing a fingerprinting approach, namely HALICDB. Data were classified by using a DL algorithm. Their obtained results proved that their proposed system gives highly accurate results as compared to conventional ML methods. We have summarized the above indoor positioning methods, as shown in Table 1.

## 3. Material and Methods

### 3.1. Locating Transmitters

Communication of BLE is comprised of connecting and advertising. Advertising is a one-way detection method. Devices that are required to be detected can transmit data packets in periods of 20 to 2000 ms. Signals are not regularly broadcasted in intervals by beacons. The higher stability of signals and highly accurate approximation are offered by shorter periods. But it affects the life of the battery. Beacon locations are determined in BLE-based techniques as signals of Bluetooth are continuously detected by the client device. Signal strength might be affected by several barriers like furniture and people and so forth. A ranging process is used to estimate the distance of the device and beacon that causes four states of proximity.

- Immediate: This state indicates the close presence of a device to beacon.
- Near: Indicates that the device is 1–3 m away from the beacon without any barrier.
- Far: It indicates low confidence due to greater distance and the presence of several barriers between the device and beacon.
- Unknown: It indicates that transmitter proximity cannot be detected.

The received signal’s strength reached by mobile phone is showed by utilizing the value of RSSI. Signal strength measured from a distance of 1 m from the device is represented by the power value of $T^{X}$. As signal strength changes with device distance, it is critical to calculating the precision of the estimated power value of $T^{X}$. The difference can be calculated by utilizing the following equation:

(1) $$d=10^{(T^{X}-RSSI)/10n},$$

here *d* represents distance, $T^{X}$ represents obtained power value of the signal from 1 m, RSSI represents the received signal’s strength, path loss index represented by *n*. It can be assigned a value depending upon the location of surroundings and beacon-like that:

2 to large wide rooms, 1.4–1.9 to corridors, 3 to rooms with furniture, 4 to rooms filled with furniture, and 5 to various floors. The following equation can be used to calculate the exponent value of path loss.

(2) $$n=-\left(\frac{RSSI-T^{X}}{10\log_{10}d\;}\right).$$

### 3.2. System Architecture

**Figure 1:**
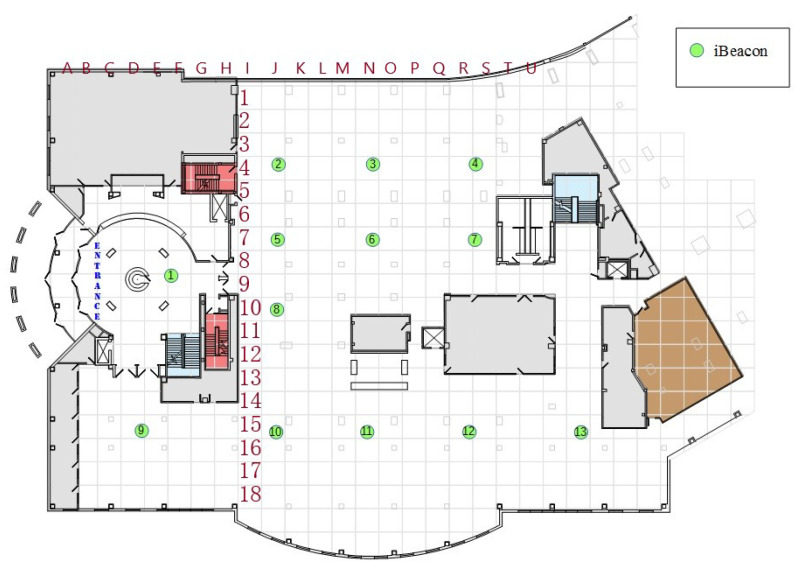
Standard building environment with the indoor localization of Beacons.

Figure 1 represents the setting of the conducted experimentation for indoor localization and its feasible implementation in a building.

**Figure 2:**
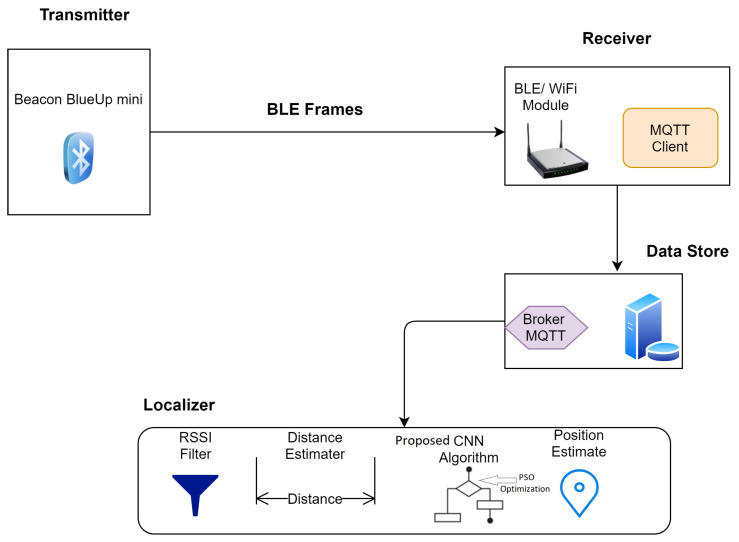
Proposed architecture of the defined localization system.

It was supposed that the BLE device will be equipped to the individual to be tracked from that particular BLE frames will be transmitted after a constant interval of time. The strength of RSSI is measured to gain the relevant messages of BLE. We implemented every receiver of BLE with a client of MQTT that renders gathered data to the MQTT receiver. Data is then forwarded to the server by the receiver, and the stored data is processed. The primary architecture of the defined localization system is given in Figure 2.

#### Proposed Framework

This research aims to use iBeacon’s Bluetooth to locate an individual in a building efficiently. This research has different potential applications, like providing a mobile to individual in a store, making sure that users do not reach selected areas, tracking pedestrian traffic and flow patterns, and several other innovative applications. In previous studies, the authors used to consider separate machine and deep learning models to predict *x* and *y* coordinates. The traditional positioning algorithm is shown in Algorithm 1. We overcome this issue by predicting both coordinates through a single model. The validation technique is the secondary issue, which we addressed in the proposed model. The employed data are taken from time series process, rotating the Bluetooth device across the building while taking a moment of about three seconds at every coordinate and taking a mean value for each cycle. The obtained mean value leads to the random split and shuffles before the predictions of both *x* and *y* points. We tried to introduce a logical split where the estimation of the last 20 percent of the data were predicted.
**Algorithm 1** Pseudo Code for basic localization system.**Require:** Record RSSI signals and number of beacons   **Ensure:** Sensor with rich RSSI value   
1:Object Beacon-detected   2:Id,   3:Major,   4:Minor,   5:MeasurementsRSSI[],   6:AverageSignalStrength   7:**if** Scan Timing != 0 **then**8: init BeaconsList[BeaconDetected]9:**end if**  10:init Array[Signals]   11:**while** Scan period not end **do**12: **if** beacon determined **then**13:   **if** beacon previously determined **then**14:     Update MeasurementsRSSI array of beacon   15:   **end if**  16:   Transfer data to Array[Signals]   17: **end if**  18: call Scan_beacon   19: **if** Array[Signals]!= 0 **then**20:   **Return** sensor data with rich RSSI value   21: **end if**  22: **Return Null**   23:**end while** 


Furthermore, we decided to transform previous models based on MLP to CNN. In the case of neural networks, that is, MLP, the network considers all 13 sensors and then generates various input neurons to every potential value of that sensor. So this implies the dealing 13 number of potential input neuronal measurements but these readings may not contain sufficient transfer value. Hence this phenomenon will lead to the result where zero information will be transferred. By transforming the input data into an image instead of a very deep matrix, CNN provides the solution. We used each, *y* representing a 10 × 10 m matrix as shown in the image given in Equation (3). Furthermore, we incorporated the sensors’ locations manually. If the value of the sensors increased, the subsequent array value will also increase.

(3) $$I\;\left(x,y\right)=\left[\begin{array}{ccc} S1 & 5 & 9\\ S2 & 9 & 14\\ S3 & 13 & 14 \end{array}\right].$$

**Figure 3:**
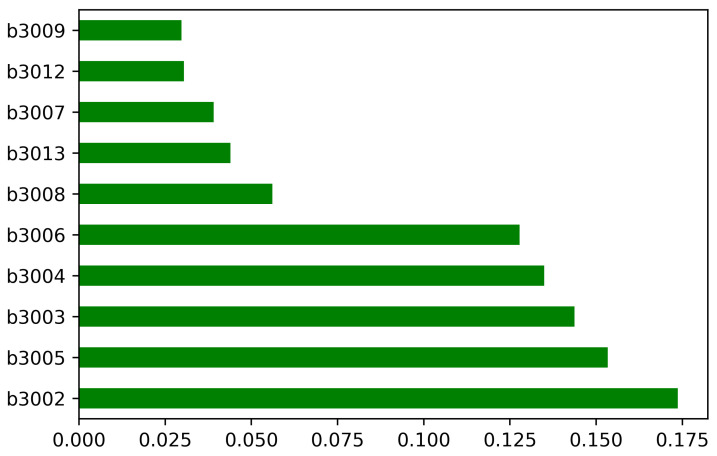
Histograms for each beacon device showing the signal strengths in terms of feature importance.

In the above equation, 1st column representing sensors, where the second column shows *x* coordinate and the third column can be interpreted as *y* coordinate. Each individual matrix for each sensor $S_{n}\left(x,y\right)$. In this way, we communicate with each 10 × 10 matrix holding the spatial coordinate information like a pixel, indicating the potential change of a sensor, adding a sensor and removing a sensor. The trend of each sensor used for the experimentation can be three possible situations, either sensor shows maximum, low or no signal. There are rare cases that any sensor produces no signal. To examine the possibility of outliers in the sensor values, histograms for each beacon device showing the signal strengths in terms of feature importance are given in Figure 3.

**Figure 4:**
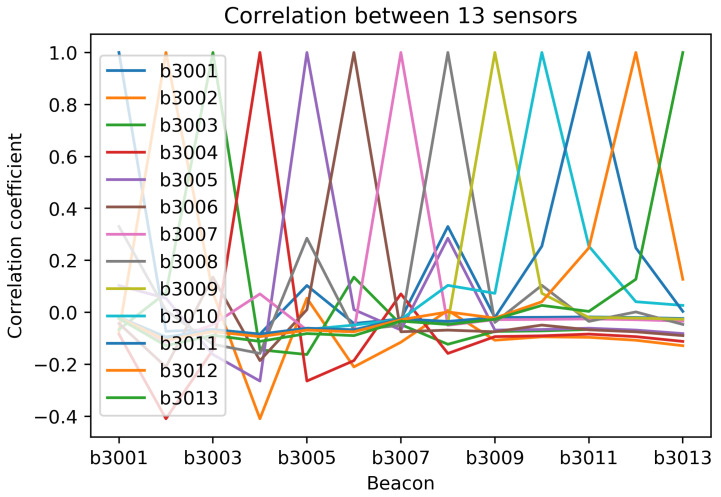
Signal’s correlation analysis of each beacons device.

Increased levels mean better signal and −200 represents the minimum signal, indicating that there is essentially no link. We also performed signal’s correlation analysis of each sensor to shows the possible measure of strength of each sensor presented in Figure 4.

We adopted a neuro-evolution approach to optimize and dynamically construct several layers in the network via enhanced PSO to establish CNN. The best global solution obtained by PSO is given directly to the weights of each layer of CNN for optimization. Besides, we used dynamic inertia weights in the PSO instead of constant inertia weight to maintain that the length of the CNN layers corresponds to the RSSI signals.

Let’s take an input image of 2-d matrix I(x,y) for each sensor $S_{n}\left(x,y\right)$ where $n=\left\{1,2,3\cdots 13\right\}$. I(x,y) is given to semi-standard CNN with 3 convolution layers 4 transpose of convolution layers, and 2 Max pooling layers with auto encoder to compress the signal readings. The equation for the input layer of auto encoder CNN can be written as:

(4) $$S_{i}={Input}_{I\left(t\right)}\left(x,y\left|D\right.\right),$$

where *t* represents the corresponding iteration, *x* and *y* are both coordinates recorded from each sensor $S_{n}\left(x,y\right)$, where $n=\left\{1,2,3\cdots 13\right\}$ and *D* shows the total dimensions of the input matrix. For each $S_{n}\left(x,y\right)$, the feature maps are calculated over the kernel window *k* using the following equation.

(5) $$F_{m}^{S_{n}}=Conv\left(S_{i},K_{s},f\left(a\right)\right).$$

In the above equation, $S_{i}$ is the actual input matrix extracted from $S_{n}\left(x,y\right)$, $K_{s}$ donates as kernel size which was set to (3,3) for each convolutional layer and $f\left(a\right)$ represents the activation function. We use ReLu function for the activation of each signal neuron in the input ${Input}_{I\left(t\right)}\left(x,y\left|D\right.\right)$. The convolutional process can be interpreted using the formula below:

(6) $$Conv\leftarrow S_{i}\times K_{s}\left[\left(x,y\left|D\right.\right)\right],S_{i}\left(x-1,y-1\right),$$

where x−1,y−1 indicating the index of each coordinate positioned by $S_{n}\left(x,y\right)$ in the targeted building. $F_{m}^{S_{n}}$ further transfer to the fully connected layer to decide the number of target classes and can be defined as:

(7) $$Y_{S_{n}}=\;F_{c}\left(Conv\leftarrow S_{i}\times K_{s}\left[\left(x,y\left|D\right.\right)\right],S_{i}\left(x-1,y-1\right)+Conv\left(S_{i},K_{s},f\left(a\right)\right)\right).$$

$Y_{S_{n}}$ contains the number of classes defined by fully connected function $F_{c}$. At this stage we employed improved PSO to tune the number of hyper parameters and to decide the evolution process among the weights of each layer. The whole optimization process is performed before the compilation of the entire model using deep features extracted from fully connected layer $Y_{S_{n}}$.

PSO is population based optimization algorithm used for optimization in several applications. For the optimization of neural networks, let us takes a population with *n* particles $P_{i}\;=\;\left\{1,2,3\cdots n\right\}$. Each particle is bound to initialize into the 2 dimensional search space of size I(x,y). The initialization processes can be described as:

(8) $$P_{i}^{t}=P_{min}^{t}+\left(P_{max}^{t}-P_{min}^{t}\right)\cdot R,$$

where *t* shows current iteration number $P_{max}^{t}$ is maximum range of search space and $P_{max}^{t}$ minimum range of search space. *R* is the random numbers over the interval of [0,1]. Each particles $P_{i}\;=\;\left\{1,2,3\cdots n\right\}$ update their current velocity $V_{i}^{t}$ and current position $X_{i}^{t}$. Both can be defined using the following two equations:

(9) $$V_{i}^{t+1}=w\cdot V_{i}^{t}+c1\cdot r1+\left(P_{\mathrm{best}}^{t}-X_{i}^{t}\right)+c2\cdot r2+\left(G_{\mathrm{best}}^{t}-X_{i}^{t}\right)$$

(10) $$X_{i}^{t+1}\;=\;{(X}_{i}^{t}+V_{i}^{t+1}).$$

In the above equations $V_{i}^{t}$ is the current velocity of each particle $P_{i}\;=\;\left\{1,2,3\cdots n\right\}$, $X_{i}^{t}$ is the current position of each particle $P_{i}\;=\;\left\{1,2,3\cdots n\right\}$, w is inertia weight factor, which is equal to 0 or any constant in the conventional PSO and hence leads to the premature convergence, c1,c2 are learning factors set to the any constant in the original PSO where r1,r2 are random numbers over the interval of [0,1]. Each individual in the multi-dimensional search area learns using their personal best solution $P_{\mathrm{best}}^{t}$ and the global best solution $G_{\mathrm{best}}^{t}$ of each swarm in the entire population. To maintain the balance among the convergence rate of each particle we proposed dynamic inertia weight factor $w_{S_{n}\left(x,y\right)\;}$ which takes signal reading of each beacons and decide the controlling factor based on the signal strength of each reading matrix I(x,y). The beacon with low signal rate will bound each particle $P_{i}\;=\;\left\{1,2,3\cdots n\right\}$ to converge with small jumps where the beacons with high signal rate will tends to increase the convergence rate which helps in improving the local best solution. The proposed inertia weight factor $w_{S_{n}\left(x,y\right)\;}$ can be represented by the following equation.

(11) $$w_{S_{n}\left(x,y\right)\;}=\;P_{min}^{t}+\left(P_{max}^{t}-P_{min}^{t}\right)\cdot {Input}_{I\left(t\right)}\left(x,y\left|D\right.\right).$$

Similarly in the proposed PSO, we replaced c1,c2 with random number generator following the uniform distribution as:

(12) $$c_{p}1,c_{p}2=\;P_{min}^{t}+\left(P_{max}^{t}-P_{min}^{t}\right)\cdot \mathrm{Rand}\left(0,1\right).$$

The updated velocity and position equation for the improved PSO will be as follows:

(13) $$V_{S_{n}}^{t+1}\;=\;w_{S_{n}\left(x,y\right)\;}\cdot V_{i}^{t}+c_{p}1\cdot r1+\left(P_{\mathrm{best}}^{t}-X_{i}^{t}\right)+c_{p}2\cdot r2+\left(G_{\mathrm{best}}^{t}-X_{i}^{t}\right)$$

(14) $$X_{S_{n}}^{t+1}\;=\;{(X}_{i}^{t}+V_{S_{n}}^{t+1}).$$

The global solution $G_{\mathrm{best}}^{t}$ obtained using improved PSO are further used in the optimization of the last layer of auto-encoder CNN for the optimization such as:

(15) $$Y_{P_{n}}=\;F_{c}(Conv\leftarrow S_{i}\times K_{s}\left[\left(x,y\left|D\right.\right)\right]+S_{i}G_{\mathrm{best}}^{t},$$

where $S_{i}G_{\mathrm{best}}^{t}$ represents the updated sensor reading based on the optimization through improved PSO. Prime steps for proposed indoor positioning are given in Algorithm 2.
**Algorithm 2** Pseudo Code for proposed indoor positioning**Require:** Record RSSI signals and number of beacons   **Ensure:** Sensor with rich RSSI value   
1:Object Beacon-detected   2:Id,   3:Major,   4:Minor,   5:MeasurementsRSSI[],   6:AverageSignalStrength   7:**if** Scan Timing != 0 **then**8: init BeaconsList[BeaconDetected]   9:**end if**  10:Convert Beacons signals into 10 * 10 I(x,y) for each sensor $S_{n}\left(x,y\right)$   11:**while** Scan period not end **do**12: **if** beacon determined **then**13:   **if** beacon previously determined **then**14:     Update ${Input}_{I\left(t\right)}\left(x,y\left|D\right.\right)$   15:     Convene each 2-d image as: $Conv\leftarrow S_{i}\times K_{s}\left[\left(x,y\left|D\right.\right)\right],S_{i}\left(x-1,y-1\right)$   16:     Initialize PSO   17:     Update velocity using $V_{S_{n}}^{t+1}=w_{S_{n}\left(x,y\right)\;}\cdot V_{i}^{t}+c_{p}1.r1+\left(P_{\mathrm{best}}^{t}-X_{i}^{t}\right)+c_{p}2.r2+$${(G}_{\mathrm{best}}^{t}-X_{i}^{t})$   18:     Update Position using $X_{S_{n}}^{t+1}={(X}_{i}^{t}+V_{S_{n}}^{t+1})$   19:   **end if**  20:   Transfer data to Array[Signals]   21:   Optimize Array[Signals] using $Y_{P_{n}}=F_{c}(Conv\leftarrow S_{i}\times K_{s}\left[\left(x,y\left|D\right.\right)\right],S_{i}G_{\mathrm{best}}^{t}$   22: **end if**  23: call Scan_beacon   24: **if** Array[Signals]!= 0 **then**25:   **Return** sensor data with rich RSSI value   26: **end if**  27: **Return Null**  28:**end while**


## 4. Results and Discussion

For the experimental simulation, we have used a data set gathered by thirteen beacons in an indoor building environment. The data set is available at [50]. Data contains 13 features compromising of thirteen beacons emitting signals from different locations about total 1420 instances corresponding to each target class. Similarly, we employed unlabeled data of similar length contains −200 RSSI strength to incorporated cluster analysis.

### 4.1. BLE Signal Analysis

Sensor sequence analysis improves to determine the attributes of BLE signal transmission concerning the range in an enclosed environment from a position transmitter. Although the distribution of the signals could be influenced by the environment (e.g., due to contouring, extras, etc.), we targeted a better understanding and having more similarities from diverse conditions regarding RSSI. During the experimentation, we seemed to have no command over the indoor building conditions and complex elements included shifting workers during the experiments and interference/noise, such as medical equipment, from electronic devices, building structures, or furniture. For RSSI, we record the average value per second. We used fixed location beacons only. To capture the signal patterns from 13 directions, the location beacons were mounted on the ceiling, walls and different other locations in indoor building.

### 4.2. Localization Results

**Figure 5:**
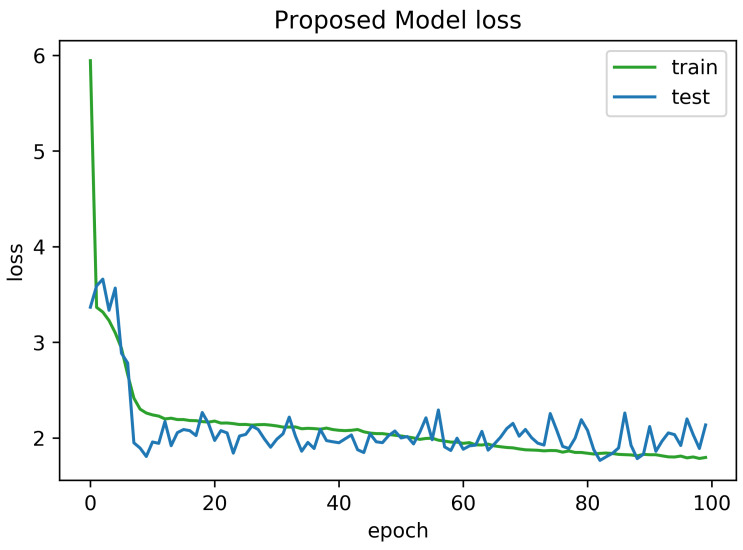
Loss curve obtained for 100 epochs using proposed algorithm with auto-encoder optimization.

**Figure 6:**
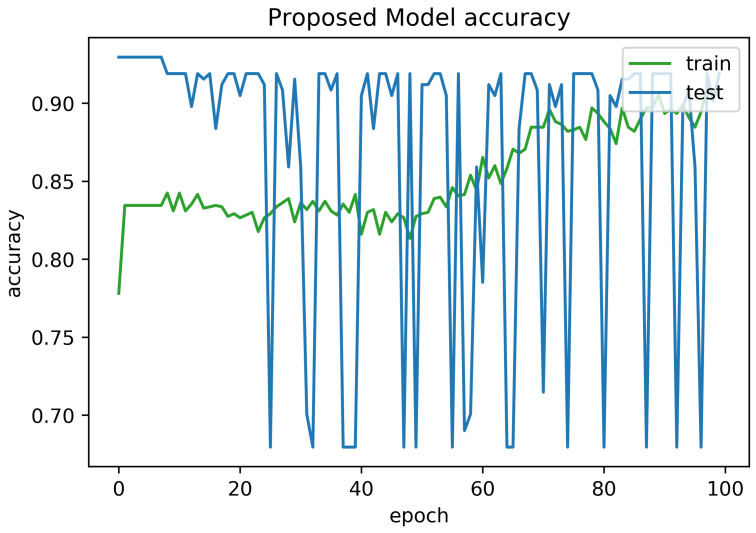
Accuracy curve obtained for 100 epochs using proposed algorithm with auto-encoder optimization.

**Figure 7:**
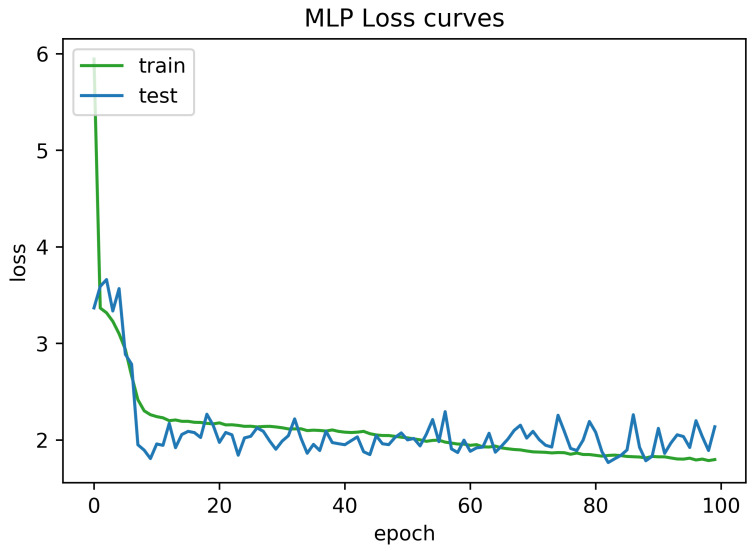
Loss curve obtained for 100 epochs using Multi Layer Perceptron (MLP) without optimization.

**Figure 8:**
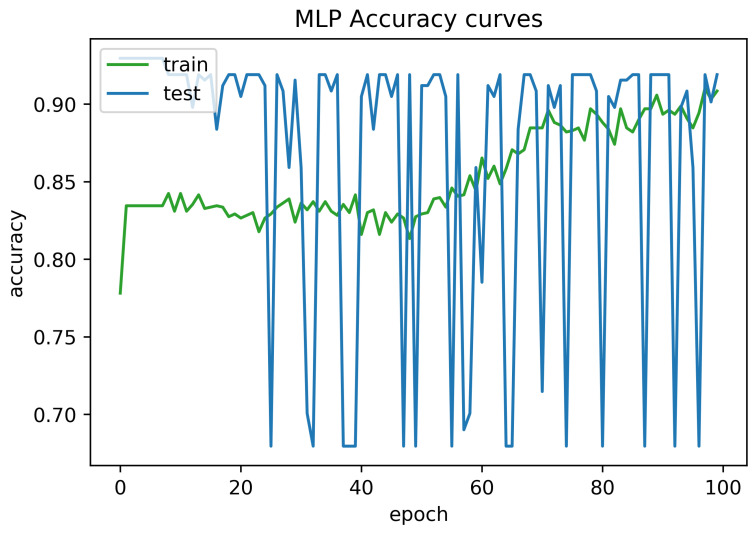
Accuracy curve obtained for 100 epochs using MLP without optimization.

To determine the frequency of BLE detectors and the location to be installed in the region to be controlled, it is necessary to analyses the real distance errors about the approximate distance between the neural network receiver and the transmitter evaluated. The distance error between the real position and the proposed estimated position is reported in Figure 5. Similarly, Figure 6 indicates the accuracy curves obtained during the training of proposed CNN for indoor localization through improved PSO. In the x-axis, it shows the number of epochs completed while receiving the RSSI signals from the thirteen installed beacons and the y-axis depicts the accuracy of localization during the validation of the proposed algorithm. Moreover, we obtained the distance error between the real position and the MLP estimated position, which can be visualized in Figure 7. The Loss curve in Figure 7 appeared a bit sloppy which is improved in the later stages when using the proposed optimized CNN as shown in Figure 5. Likewise, Figure 8 designates the accuracy curves acquired during the training of MLP to indoor localization through conventional PSO.

We selected thirteen possible positions (x, y) of the beacons in the area, to evaluate the accuracy of the position procedure, reported in Table 2. Given numerous beacons greater than or equal to thirteen in known locations, the optimization method CNN algorithm solves non-linear problems. The array with the length approximation should therefore be associated with at least thirteen features. In Table 2, we compare the proposed optimized CNN with other states of the art ML and DL algorithms to show the competency of the proposed algorithm. The comparative algorithms including Decision Tree, KNN, Logistic Regression, SVM, ANN, ANN with dropout, DNN, DNN with dropout and finally MLP. Two major evaluation metrics were used to compare the experimental results which involve Average Accuracy and Error Rate (referred to Table 2). Average Accuracy shows the percentage of accurate localization from thirteen target classes where the Error rate can be defined as 1-Accuracy.

**Figure 9:**
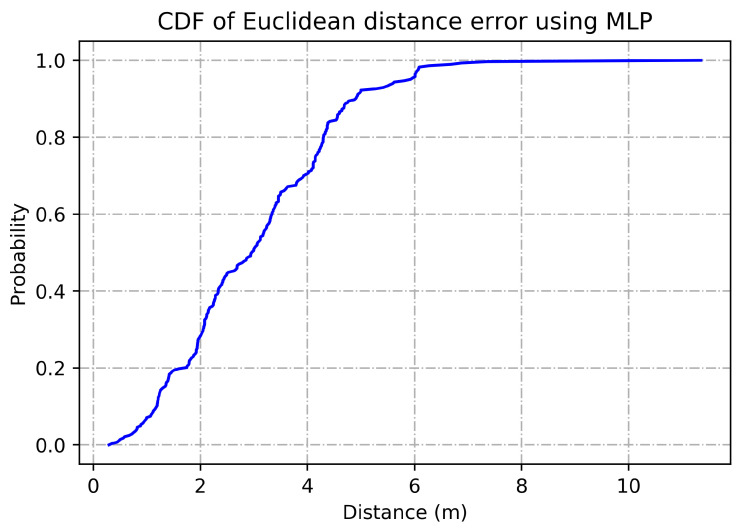
CDF of Euclidean distance error using MLP.

**Figure 10:**
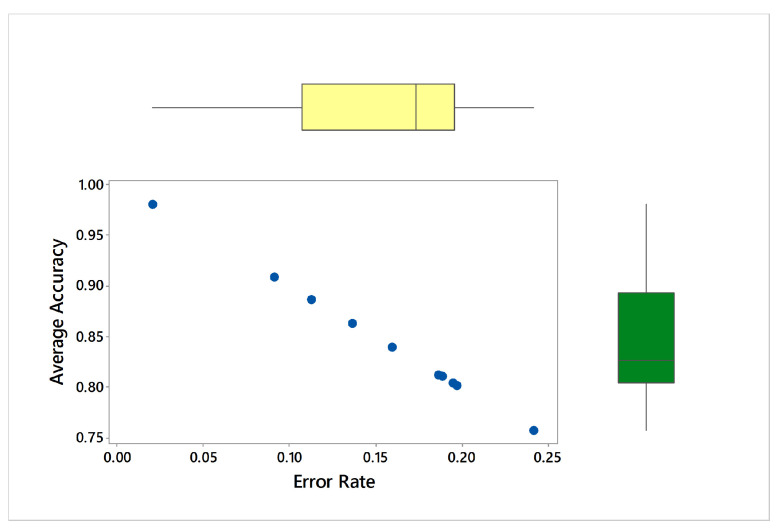
Marginal correlation analysis between error and accuracy obtained through thirteen Bluetooth low energy (BLE) beacons.

Figure 9 represents CDF of Euclidean distance error using MLP. The X-axis indicates the distance in meters from the beacons with no greater than 200 range and no lesser than −200 range where Y-axis shows the probability of accurate indoor positioning. For the validation of conducted experiments, we have performed statistical tests on the obtained results. Table 3 represents Kruskal-Wallis Test: Average accuracy versus Models where Table 4 shows Kruskal-Wallis Test: Error rate versus Models. In case of accuracy comparison, the best algorithm should have a higher Kruskal-Wallis rank while in case of loss estimation the best algorithm should have a lower Kruskal-Wallis rank. Likewise, Figure 10 visualizes marginal correlation analysis between error and accuracy obtained through thirteen BLE beacons. We can see that marginal correlation of error rate from 0.00 to 0.25 tends to decrease which mean that the more signal strength appears to be strong, the more error rate will be decreased.

### 4.3. Analysis

The experimental analysis reveals that an increase in the thirteen beacons considered (and so in the approximate ranges) leads to high errors in the estimation of the position. This implies that the beacon signals with large loss affect the reliability of the process.

We have interrupted beacons to be labeled under the RSSI (best case) limit, thereby preventing the localizer from using its estimation sets. We replicated the measurements to test these cases by removing the beacons with the lowest feature vector provided by the Decision Tree classifier. In the instance of a vector of one obstructed function (i.e., 7.6 percent, since the proposed optimized CNN algorithm considers the best thirteen estimates). According to the order in which they appeared, we were expected to remove another of the thirteen best receivers. For the top ten best sensors and the thirteen transmit positions recorded, we summed this process.

We can observe the best performance of the proposed algorithm from Table 2, with 97.92% average accuracy. After the proposed CNN, MLP performed well and appeared as the second-best performer with 90.85% accuracy. The difference ratio between proposed and MLP can be described as the number of total correct predictions using proposed CNN minus the number of total correct predictions using MLP divided by the summation of the total number of wrong predictions using both proposed and MLP.

The worst performance in terms of low average accuracy and the high error rate is obtained by KNN with (75.78% average accuracy and 24.22% error rate). Similarly, error rate between 0.1 to 0.2 for each RSSI beacons were recorded for Decision Tree (0.1605), Logistic Regression (0.1368), SVM (0.1133), ANN (0.1873) and DNN (0.1890) respectively.

From Table 3 and Table 4, we can see the highest Kruskal-Wallis rank of proposed optimized CNN with 10.0 and highest Z-score of 1.57 proves the superiority of the proposed algorithm statistically. Similarly, proposed CNN obtained the lowest Kruskal-Wallis rank (1.0) in the case of error rate with Z-score is negative −1.57.

We also observed that in some cases, the removal of inappropriate signals lead to missing location data for updating the position coordinate, such as when only the signals from wrong directions were detected but the participant’s object and structural elements were obscured from the right location. Although this was planned, we did not attempt to optimize signal sensing or calculating the strategic positioning of location beacons. To ensure a proper connection between the location beacons and the RSSI signal strength, we placed the location observatories on the walls and floor; however, we did not equate the reliability of the direction detectors installed on the other locations.

## 5. Conclusions

BLE beacons have been used to map individuals’ locations in indoor environments. Devices need to be programmed to allow for complex patterns of interference when tracking indoor positions using the RSSI. Our proposed framework based on optimized CNN provides an robust method for tracking the indoor location of a moving user using BLE beacons in complex indoor building environments. We developed a 2D image-based CNN positioning model consisting of the number of signals obtained from both the x and y-axis indicators. The best global solution obtained by PSO is given directly to the weights of each layer of CNN for optimization of CNN. Besides, instead of constant inertia weight, we used dynamic inertia weights in the PSO to maintain that the CNN layer length corresponds to BLE’s RSSI signals. Experiments are performed in a building environment in which thirteen beacon systems were mounted to record coordinates at various locations. We further used ML and DL methods for predicting the position of a subject in an indoor environment for assessment comparison. The experimental results show that, relative to other recent approaches, the proposed optimized CNN-based approach demonstrates high precision for monitoring the locations of a moving consumer in a complex building without complex calibration.

In future work, we intend to use other meta-heuristic models such as Bat Algorithm and Differential Evolution Algorithm to improve the current performance with more complex indoor architecture.

## Figures and Tables

**Table 1:** Brief Description of recent related studies on indoor positioning using several machine and deep learning approaches.

Ref	Data Set	Sensors/ Device	Model	Results
[27]	RSSI	Bluetooth	RSSI	The RNSI-based method gained accuracy (80.0%)
[28]	RSSI, SNR	Bluetooth device	feed-forward neural network	The method showed an accuracy of estimated position under 1 m
[29]	RSSI	Bluetooth	Neural network	Obtained high accuracy
[30]	RSSI	Bluetooth	RFID	Showed promising results
[31]	RSS	Wi-Fi fingerprints, iBeacons, and sensors of smartphone	k-nearest neighbour (KNN) algorithm	Showed maximum error of 2.77 m
[32]	UWB	UWB	IPSs	Obtained good performance
[35]	CRLB	Wi-Fi, Bluetooth	Motley-Keenan model	Gained efficient results
[36]	RSSI	Smart phone	Dense Neural Network	Showed 95% accurate results
[37]	LPWA	Wi-Fi	multilayer neural network (MNN)	Gained 98% accurate results
[38]	RSS	WiFi, ZigBee or Bluetooth devices	k-nearest neighbor	predicted error in position was about 1.8 m
[39]	RSSI	Mobile phone	Expectation-maximization (EM)	Showed good localization performance
[40]	RSSI	Bluetooth	fuzzy logic	Gained highly accurate results
[41]	RSSI	mobile device	log-normal distance path loss (LDPL)	Showed localization error less than 2.5 m
[42]	DR	Smart phone	lon-short term memory (LSTM)	distance error of <2.4% and >1.5%
[43]	RSSI	Bluetooth	WD-based GSF framework	Showed better results than conventional methods
[44]	Wi-Fi RTT	Wi-Fi	Particle filters	Gained efficient results
[45]	RSSI	Bluetooth	fuzzy logic	Showed good performance
[46]	RSSI	Fingerprint	k-nearest neighbors (k-NN) algorithm	provided useful predictions
[48]	RSS	Bluetooth	Centroid Approximation (CA) and Minimum Mean Square Error (MMSE)	Showed highly precise results
[49]	HALICDB	Fingerprint	Stacked sparse autoencoder	Obtained high accuracy

**Table 2:** Accuracy and error rate of detecting indoor location using thirteen beacons through state of the art ML and DL algorithms.

Model	Average Accuracy	Error Rate
Decision Tree	0.8395	0.1605
KNN	0.7578	0.2422
Logistic Regression	0.8632	0.1368
SVM	0.8867	0.1133
ANN	0.8127	0.1873
ANN_Dropout	0.8021	0.1979
DNN	0.8110	0.1890
DNN_Dropout	0.8047	0.1953
MLP	0.9085	0.0915
CNN	0.9135	0.0865
Proposed	**0.9792**	**0.0208**

**Table 3:** Kruskal-Wallis Test: Average accuracy versus Models.

Model	Median	Rank	Z−Score
ANN	0.8127	5.0	−0.17
ANN_Dropout	0.8021	2.0	−1.22
Decision Tree	0.8395	6.0	0.17
DNN	0.8110	4.0	−0.52
DNN_Dropout	0.8047	3.0	−0.87
KNN	0.7578	1.0	−1.57
Logistic Regression	0.8632	7.0	0.52
MLP	0.9085	9.0	1.22
SVM	0.8867	8.0	0.87
CNN	0.9135	9.0	1.27
Proposed	0.9792	10.0	1.57

**Table 4:** Kruskal-Wallis Test: Error rate versus Models.

Model	Median	Rank	Z−Score
ANN	0.18730	6.0	0.17
ANN_Dropout	0.19790	9.0	1.22
Decision Tree	0.16050	5.0	−0.17
DNN	0.18900	7.0	0.52
DNN_Dropout	0.19530	8.0	0.87
KNN	0.24220	10.0	1.57
Logistic Regression	0.13680	4.0	−0.52
MLP	0.09150	2.0	−1.22
SVM	0.11330	3.0	−0.87
CNN	0.01865	2.0	−1.27
Proposed	0.02080	1.0	−1.57
